# Prediction of Isolated Local Recurrence After Resection of Pancreatic Ductal Adenocarcinoma: A Nationwide Study

**DOI:** 10.1245/s10434-024-15664-4

**Published:** 2024-06-27

**Authors:** I. W. J. M. van Goor, P. C. M. Andel, F. S. Buijs, M. G. Besselink, B. A. Bonsing, K. Bosscha, O. R. Busch, G. A. Cirkel, R. M. van Dam, S. Festen, B. Groot Koerkamp, E. van der Harst, I. H. J. T. de Hingh, G. Kazemier, M. S. L. Liem, G. Meijer, V. E. de Meijer, V. B. Nieuwenhuijs, D. Roos, J. M. J. Schreinemakers, M. W. J. Stommel, F. Wit, R. C. Verdonk, H. C. van Santvoort, I. Q. Molenaar, M. P. W. Intven, L. A. Daamen, The Dutch Pancreatic Cancer Group

**Affiliations:** 1 Department of Surgery, Regional Academic Cancer Center Utrecht, Utrecht University, University Medical Center Utrecht Cancer Center & St. Antonius Hospital Nieuwegein, Utrecht, The Netherlands; 2 Department of Radiation Oncology, Regional Academic Cancer Center Utrecht, Utrecht University, University Medical Center Utrecht Cancer Center, Utrecht, The Netherlands; 3 Department of Surgery, Amsterdam UMC, University of Amsterdam, Amsterdam, The Netherlands; 4 Cancer Center Amsterdam, Amsterdam, The Netherlands; 5 Department of Surgery, Leiden University Medical Center, Leiden, The Netherlands; 6 Department of Surgery, Jeroen Bosch Hospital, Den Bosch, The Netherlands; 7 Department of Medical Oncology, University Medical Center Utrecht Cancer Center & Meander Medical Center Amersfoort, Regional Academic Cancer Center Utrecht, Utrecht University, Utrecht, The Netherlands; 8 Department of Surgery, Maastricht University Medical Center+, Maastricht, The Netherlands; 9 Department of Surgery, OLVG, Amsterdam, The Netherlands; 10 Department of Surgery, Erasmus Medical Center Cancer Institute, Rotterdam, The Netherlands; 11 Department of Surgery, Maasstad Hospital, Rotterdam, The Netherlands; 12 Department of Surgery, Catharina Hospital, Eindhoven, The Netherlands; 13 Department of Surgery, Amsterdam University Medical Center, Vrije Universiteit, Amsterdam, The Netherlands; 14 Department of Surgery, Medisch Spectrum Twente, Enschede, The Netherlands; 15 Department of Surgery, University of Groningen, University Medical Center Groningen, Groningen, The Netherlands; 16 Department of Surgery, Isala, Zwolle, The Netherlands; 17 Department of Surgery, Renier de Graaf Gasthuis, Delft, The Netherlands; 18 Department of Surgery, Amphia Hospital, Breda, The Netherlands; 19 Department of Surgery, Radboud University Medical Center, Nijmegen, The Netherlands; 20 Department of Surgery, Tjongerschans Hospital, Heerenveen, The Netherlands; 21 Department of Gastroenterology, Regional Academic Cancer Center Utrecht, Utrecht, The Netherlands; 22 Imaging Division, University Medical Centre Utrecht, Utrecht University, Utrecht, The Netherlands

## Abstract

**Background:**

Distinguishing postoperative fibrosis from isolated local recurrence (ILR) after resection of pancreatic ductal adenocarcinoma (PDAC) is challenging. A prognostic model that helps to identify patients at risk of ILR can assist clinicians when evaluating patients’ postoperative imaging. This nationwide study aimed to develop a clinically applicable prognostic model for ILR after PDAC resection.

**Patients and Methods:**

An observational cohort study was performed, including all patients who underwent PDAC resection in the Netherlands (2014–2019; NCT04605237). On the basis of recurrence location (ILR, systemic, or both), multivariable cause-specific Cox-proportional hazard analysis was conducted to identify predictors for ILR and presented as hazard ratios (HRs) with 95% confidence intervals (CIs). A predictive model was developed using Akaike’s Information Criterion, and bootstrapped discrimination and calibration indices were assessed.

**Results:**

Among 1194/1693 patients (71%) with recurrence, 252 patients (21%) developed ILR. Independent predictors for ILR were resectability status (borderline versus resectable, HR 1.42; 95% CI 1.03–1.96; *P* = 0.03, and locally advanced versus resectable, HR 1.11; 95% CI 0.68–1.82; *P* = 0.66), tumor location (head versus body/tail, HR 1.50; 95% CI 1.00–2.25; *P* = 0.05), vascular resection (HR 1.86; 95% CI 1.41–2.45; *P* < 0.001), perineural invasion (HR 1.47; 95% CI 1.01–2.13; *P* = 0.02), number of positive lymph nodes (HR 1.04; 95% CI 1.01–1.08; *P* = 0.02), and resection margin status (R1 < 1 mm versus R0 ≥ 1 mm, HR 1.64; 95% CI 1.25–2.14; *P* < 0.001). Moderate performance (concordance index 0.66) with adequate calibration (slope 0.99) was achieved.

**Conclusions:**

This nationwide study identified factors predictive of ILR after PDAC resection. Our prognostic model, available through www.pancreascalculator.com, can be utilized to identify patients with a higher a priori risk of developing ILR, providing important information in patient evaluation and prognostication.

**Supplementary Information:**

The online version contains supplementary material available at 10.1245/s10434-024-15664-4.

Pancreatic ductal adenocarcinoma (PDAC) is characterized by a poor prognosis and currently represents the fourth leading cause of cancer-related death.^1^ The best chances of survival are achieved through radical resection combined with (neo)adjuvant systemic therapy.^2–4^ Nevertheless, the majority of patients develop disease recurrence within 2 years after surgery, causing a disappointing 5-year survival rate of 17%.^5,6^

About a quarter of patients with disease recurrence after resection of PDAC develop isolated local recurrence (ILR).^7^ The prognosis of these patients is superior compared with patients with disease recurrence in the liver or at multiple sites, reflected by a median overall survival (OS) of 26 months versus 15 months and 18 months, respectively.^7^ As previously suggested, ILR might arise from residual microscopic tumor deposits in the pancreatic remnant or surrounding tissues, which lack the capability to survive at distant sites.^7^ This is considered to contribute to the relatively favorable prognosis observed in patients experiencing ILR.

The distinctive tumor biology of ILR, characterized by a less aggressive nature and a propensity for slower growth, might be more amenable to control through localized ablative treatment interventions (e.g., stereotactic radiotherapy). However, identification of ILR poses considerable challenges for healthcare professionals. Distinguishing postoperative fibrosis from disease recurrence in the pancreatic remnant or surgical bed is a difficult task, which often makes repetitive imaging necessary to confirm or negate the diagnosis of disease recurrence.^8,9^

Prognostic factors could be helpful to identify patients at risk of developing ILR after PDAC resection. However, studies investigating ILR-specific risk factors have reported conflicting results. For instance, a positive resection margin status (R1 < 1 mm, defined as presence of tumor cells within 1 mm of the resection margin) was associated with ILR in one study but not in two others, whilst one of those did show an association with R1 direct (tumor cells directly involved in the resection margin).^5,10,11^ Adjuvant chemoradiotherapy was associated with a reduced likelihood of ILR, but contradictory results were published regarding adjuvant chemotherapy.^5,11^ Moreover, nodal status (N1 and N2 compared with N0) was found to be associated with ILR.^10^ As reflected by these discrepancies, further clarification of factors predictive of ILR is desired. In addition, development of a clinically applicable prognostic model that incorporates the optimal combination of factors predictive of ILR might provide additional insights and has not been done before. Such a model has the potential to enable identification of patients with a higher a priori risk of developing ILR and might assist healthcare professionals when evaluating patients’ postoperative imaging.

Therefore, the aim of this study was to identify factors associated with ILR of PDAC and to develop a clinically applicable prognostic model with the best predictive ability.

## Patients and Methods

### Patient Selection

All Dutch centers performing pancreatic cancer surgery participated in this nationwide, observational cohort study (NCT04605237). The scientific committee of the Dutch Pancreatic Cancer Group (DPCG) has approved this study.^12^ In all participating centers, institutional board approval was obtained. Patients who underwent resection of PDAC between 2014 and 2019, as registered in the prospective, mandatory Dutch Pancreatic Cancer Audit (DPCA), were eligible.^13^ In case of complication-related mortality within 90 days after surgery, a macroscopically irradical resection (R2), or unknown resection margin status, patients were excluded. Patients with unknown recurrence status or location were also excluded. The Transparent Reporting of a multivariable prediction model for Individual Prognosis or Diagnosis (TRIPOD) and the Strengthening the Reporting of Observational Studies in Epidemiology (STROBE) guidelines were followed.^14,15^

### Data Collection and Predictor Selection

Baseline and perioperative data were retrieved from the DPCA. Data regarding pathological features, follow-up, disease recurrence, and survival were additionally collected from the electronic patient records in each participating hospital.

Resectability status was defined according to DPCG criteria.^16^ (Neo)adjuvant therapy was considered completed if at least 80% of the planned number of cycles was received by the patient. During the study period, neoadjuvant chemo(radio)therapy was only given in the context of a clinical trial and the planned number of cycles therefore depended on the study protocol.^17,18^ Adjuvant strategies where unified in all participating institutions according to the Dutch national guidelines.^19^ Within the Netherlands, radiotherapy has no role in the adjuvant treatment of patients with pancreatic cancer. Postoperative complications for which surgical or radiological intervention or intensive care unit admittance were necessary or lead to single- or multi-organ failure or patient demise, were scored as major complications. Tumor (T) stage, lymph node (N) status, and tumor, node, metastasis (TNM)-status were defined according to the eighth edition of the American Joint Committee on Cancer TNM guidelines.^20^ Resection margin status was considered microscopically positive (R1 < 1 mm) if tumor cells were present within 1 mm of the closest resection margin, apart from the anterior surface.^19^

Potential predictors were selected on the basis of previously suggested associations with PDAC recurrence, including resectability status (resectable, borderline resectable, or locally advanced), completion of neoadjuvant therapy (yes or no), preoperative serum carbohydrate antigen 19-9 (CA 19-9) level (logarithmic in U/mL), location of the tumor (head or body/tail), vascular resection (yes or no), tumor size (continuous in mm), tumor differentiation (well/moderate or poor), perineural invasion (yes or no), lymphovascular invasion (yes or no), positive regional lymph nodes (continuous), resection margin status (R0 ≥ 1 mm or R1 < 1 mm), and completion of adjuvant chemotherapy (yes or no).

### Outcomes and Definitions

The primary outcome was the presence of ILR. Diagnosis of disease recurrence was preferably based on histology, but if absent, consensus from a multidisciplinary team meeting based on results of imaging and serum tumor markers sufficed. In the Netherlands, national guidelines advise follow-up on the basis of clinical symptoms after resection of PDAC.^19^ If symptoms suspicious of disease recurrence arise, imaging can be performed (symptomatic strategy). However, in the case of study participation or patient preference, imaging could have been performed at set intervals (e.g., monthly, 3-monthly, yearly), which was defined as recurrence-focused follow-up.

### Statistical Analysis

Patients were divided into three groups ono the basis of recurrence status at initial diagnosis of disease recurrence: ILR, systemic disease recurrence, or local and systemic disease recurrence. Descriptive statistics were used to present baseline characteristics. Multiple imputation with the iterative Markov chain Monte Carlo method (five imputations; ten iterations) was used for missing baseline data, which were considered missing at random.^21^ Categorical variables were shown as absolute numbers with corresponding percentages and compared via the Chi-Square test. Continuous variables were expressed as a mean ± standard deviation (SD) or median with interquartile range (IQR) and compared via an analysis of variance (ANOVA). Proportionality of predictors was examined by calculating Schoenfeld residuals, and variables were transformed in case of a nonnormal distribution. Multicollinearity between predictors was ruled out by determining variable inflation factors.^22^ Disease-free survival (DFS) and OS were determined by Kaplan–Meier survival curves and presented as median with 95% confidence intervals (95% CIs). Patients with missing survival data were excluded. DFS was defined as the time between the date of surgery and the date of recurrence diagnosis. OS was defined as the time between the date of surgery and the date of death from any cause. Patients without event were censored at the date of last follow-up.

Since development of ILR versus systemic or synchronous local and systemic recurrence were considered competing risks, multivariable cause-specific Cox proportional hazard analysis was performed to identify prognostic factors associated with ILR. Patients with disease recurrence in other locations than ILR were censored at date of recurrence diagnosis, and patients without disease recurrence were censored at the date of last follow-up. Results were presented as hazard ratios (HRs) with 95% CIs and probability values (*P*). HRs greater than 1 were associated with the development of ILR. The best predictive model was selected by Akaike’s information criterion and internally validated in 1000 bootstrap samples. The concordance index (C-index) was used to determine discriminative ability, in which perfect discrimination is reflected by a value of 1. Calibration plots with a calibration slope were constructed to assess calibration.

The HRs of predictors included in the final model were translated into risk scores. The sum of individual risk scores leads to a total score, which directly reflects the probability of an individual patient to develop ILR at a certain time point. The final model was made available as an online calculator on www.pancreascalculator.com.

R language environment was used to perform statistical analyses (version 3.3.0+, readxl, naniar, car, dplyr, tidyr, arsenal, mice, survival, survminer, rms, MASS packages; http://R-project.org). A two-sided *P*-value of less than 0.05 was considered statistically significant.

## Results

In total, 1909 patients were identified. Of those, 216 patients (11%) were excluded (Supplementary Fig. 1). Consequently, 1693 patients (89%) were included, with a median follow-up of 45 months [interquartile range (IQR) 33–60 months] and median OS of 22 months (95% CI 20–23 months; Table 1).

**Table 1:** Baseline characteristics and missing data of 1693 patients who underwent resection of pancreatic ductal adenocarcinoma

	Before imputation	Missing, *n* (%)	After imputation
Age at diagnosis, mean (SD), years	67 (9)	0 (0)	67 (9)
Male sex, *n* (%)	899 (53)	0 (0)	899 (53)
BMI, mean (SD), kg/m^2^	25 (4)	15 (1)	25 (4)
CACI, mean (SD)	3 (2)	1 (0)	3 (2)
ASA-score, *n* (%)		18 (1)	
I	177 (10)		180 (11)
II	1050 (62)		1060 (63)
III	439 (26)		444 (26)
IV	9 (1)		9 (1)
ECOG performance score at primary diagnosis, *n* (%)		436 (26)	
0	621 (37)		817 (48)
1	518 (31)		707 (42)
2	95 (6)		135 (8)
3	22 (1)		31 (2)
4	1 (0)		3 (0)
Resectability, *n* (%)		122 (7)	
Resectable	1209 (71)		1305 (77)
Borderline resectable	242 (14)		259 (15)
Locally advanced	120 (7)		129 (8)
Neoadjuvant therapy, *n* (%)		13 (1)	
None	1442 (85)		1442 (85)
FOLFIRINOX chemotherapy	156 (9)		156 (9)
Gemcitabine chemoradiotherapy	76 (5)		76 (5)
Other chemotherapy	6 (0)		6 (0)
Completed neoadjuvant therapy^a^, *n* (%)	228 (96)	0 (0)	228 (96)
Preoperative serum CA 19-9 level, median (IQR), U/mL	129 (34−479)	424 (25)	130 (34–499)
Type of surgery, *n* (%)		6 (0)	
Open	1436 (85)		1441 (85)
Laparoscopic	123 (7)		124 (7)
Robot-assisted	128 (8)		128 (8)
Surgical procedure, *n* (%)		0 (0)	
Pancreatoduodenectomy	1343 (79)		1343 (79)
Distal pancreatectomy	262 (16)		262 (16)
Total pancreatectomy	57 (3)		57 (3)
Other	31 (2)		31 (2)
Tumor location, *n* (%)		39 (2)	
Head	1356 (80)		1388 (82)
Body/tail	298 (18)		305 (18)
Vascular resection, *n* (%)	479 (28)	3 (0)	479 (28)
Tumor differentiation, *n* (%)		278 (16)	
Well/moderate	1027 (61)		1230 (73)
Poor	388 (23)		463 (27)
Microscopic lymphovascular invasion, *n* (%)	896 (53)	238 (14)	1034 (61)
Microscopic perineural invasion, *n* (%)	1275 (75)	143 (8)	1384 (82)
Tumor stage 8th AJCC edition, *n* (%)		32 (2)	
T1	227 (13)		232 (14)
T2	996 (59)		1012 (60)
T3	417 (25)		428 (25)
T4	21 (1)		21 (1)
Lymph node stage eighth AJCC edition, *n* (%)		5 (0)	
N0	513 (30)		515 (30)
N1	638 (38)		640 (38)
N2	537 (32)		538 (32)
Total resected lymph nodes, median (IQR)	15 (11–21)	15 (1)	15 (11–21)
Lymph node ratio, *n* (%)		18 (1)	
≤ 0.2	1072 (63)		1086 (64)
> 0.2	603 (36)		607 (36)
TNM stage eighth AJCC edition, *n* (%)		35 (2)	
≤ 2A	498 (29)		511 (30)
≥ 2B	1160 (69)		1182 (70)
Major postoperative complications^b^, *n* (%)	580 (34)	0 (0)	580 (34)
Resection margin status^c^, *n* (%)		0 (0)	
R0 ≥ 1 mm	882 (52)		882 (52)
R1 < 1 mm	811 (48)		811 (48)
Adjuvant chemotherapy, *n* (%)	1050 (62)	27 (2)	1064 (62)
Completed adjuvant chemotherapy^a^, *n* (%)	664 (63)	0 (0)	664 (62)
Type adjuvant chemotherapy, *n* (%)		31 (3)	
Gemcitabine monotherapy	720 (69)		720 (68)
FOLFIRINOX	155 (15)		155 (15)
Gemcitabine combination therapy	133 (13)		133 (13)
Other	11 (1)		11 (1)

Percentages may not add up to 100 because of rounding

^a^(Neo)adjuvant therapy was considered completed in cases where 80% of the planned number of cycles was received by the patient

^b^Major postoperative complications were defined as complications requiring surgical or radiologic intervention, intensive care unit admittance, single- or multi-organ failure, of the patients’ demise

^c^Resection margin status was considered microscopically positive (R1 < 1 mm) if tumor cells were present within 1 mm of the closest resection margin, apart from the anterior surface

*AJCC* American Joint Committee on Cancer, *ASA* American Society of Anesthesiologists, *BMI* body mass index, *CA 19-9* carbohydrate antigen 19-9, *CACI* Charlson age-adjusted comorbidity index, *ECOG* Eastern Cooperative Oncology Group, *FOLFIRINOX* fluorouracil, leucovorin, irinotecan, oxaliplatin, *IQR* interquartile range, *SD* standard deviation

A total of 1194 patients (71%) developed disease recurrence with a median DFS of 11 months (95% CI 11–12 months). Of those, 252 patients (21%) developed ILR. Systemic disease recurrence without local recurrence occurred in 473 patients (40%), of whom 182 patients (38%) had liver only recurrence, 86 patients (18%) had lung only recurrence, 182 patients (38%) had multiple site recurrence, and 23 patients (5%) had isolated recurrence at another distant site. Synchronous local and systemic recurrence was present in 469 patients (39%; Table 2). When comparing patients with ILR to those with synchronous local and systemic recurrence, patients with ILR more often underwent a vascular resection (39% versus 31%; *P* = 0.03), had a favorable tumor stage (*P* < 0.01), and had tumors that were more often well/moderately differentiated (76% versus 69%; *P* = 0.02) with less lymphovascular invasion (55% versus 71%; *P* < 0.001). In addition, they more often received and completed adjuvant chemotherapy (67% versus 56%; *P* < 0.01 and 71% versus 63%; *P* < 0.01, respectively). In patients with ILR, standardized follow-up imaging to detect disease recurrence was more frequently applied (22% versus 14%; *P* = 0.02; Supplementary Table 1).

**Table 2:** Descriptive statistics comparing patients with isolated local pancreatic ductal adenocarcinoma recurrence with patients with local and systemic recurrence

	ILR (*n* = 252)	Systemic (*n* = 473)	Local and systemic (*n* = 469)	*P*
Age at diagnosis, mean (SD), years	66 (10)	67 (9)	67 (10)	0.76
Male sex, n (%)	136 (54)	260 (55)	244 (52)	0.66
BMI, mean (SD), kg/m^2^	25 (4)	25 (4)	25 (4)	0.58
CACI, mean (SD)	3 (2)	3 (2)	3 (2)	0.94
Resectability, *n* (%)				0.04
Resectable	175 (70)	374 (79)	355 (76)	
Borderline resectable	56 (22)	62 (13)	81 (17)	
Locally advanced	21 (8)	36 (8)	33 (7)	
Neoadjuvant therapy, *n* (%)	42 (17)	79 (17)	66 (14)	0.47
Completed neoadjuvant therapy^a^, *n* (%)	37 (71)	72 (91)	61 (92)	0.60
Preoperative serum CA 19-9 level, median (IQR), U/mL	157 (34–514)	154 (47–499)	179 (48–593)	0.13
Tumor location, *n* (%)				< 0.001
Head	226 (90)	370 (78)	397 (85)	
Body/tail	26 (11)	103 (22)	72 (15)	
Vascular resection, *n* (%)	99 (39)	132 (28)	145 (31)	< 0.01
Tumor stage 8th AJCC edition, *n* (%)				< 0.01
T1	22 (9)	56 (12)	52 (11)	
T2	176 (70)	272 (57)	264 (56)	
T3	49 (20)	140 (29)	147 (31)	
T4	5 (2)	5 (1)	6 (1)	
Tumor differentiation, *n* (%)				0.03
Well/moderate	192 (76)	320 (68)	323 (69)	
Poor	60 (24)	153 (32)	146 (31)	
Perineural invasion, *n* (%)	217 (86)	393 (83)	405 (86)	0.30
Lymphovascular invasion, *n* (%)	139 (55)	316 (67)	334 (71)	< 0.001
Lymph node status eighth AJCC edition, *n* (%)				0.30
N0	69 (27)	119 (25)	116 (25)	
N1	103 (41)	189 (40)	168 (36)	
N2	80 (32)	165 (35)	185 (39)	
Resection margin status^b^, *n* (%)				< 0.01
R0 ≥ 1 mm	103 (41)	262 (55)	210 (45)	
R1 < 1 mm	149 (59)	211 (45)	259 (55)	
Adjuvant chemotherapy, *n* (%)	170 (67)	296 (63)	264 (56)	0.01
Completed adjuvant chemotherapy^a^, *n* (%)	120 (71)	199 (74)	166 (63)	< 0.01
Use of imaging procedures during follow-up^c^, *n* (%)				0.11
None/nonstandardized	195 (78)	376 (82)	390 (83)	
Standardized	54 (22)	84 (18)	68 (14)	

Percentages may not add up to 100 because of rounding and missing data

^a^(Neo)adjuvant therapy was considered completed in cases where 80% of the planned number of cycles was received by the patient

^b^Resection margin status was considered microscopically positive (R1 < 1 mm) if tumor cells were present within 1 mm of the closest resection margin, apart from the anterior surface

^c^Postoperative imaging could have been performed in a standardized fashion at set intervals, or when indicated by clinical symptoms

*AJCC* American Joint Committee on Cancer, *BMI* body mass index, *CA 19-9* carbohydrate antigen 19-9, *CACI* Charlson age-adjusted comorbidity index, *IQR* interquartile range, *SD* standard deviation

### Survival Estimates

**Fig. 1:**
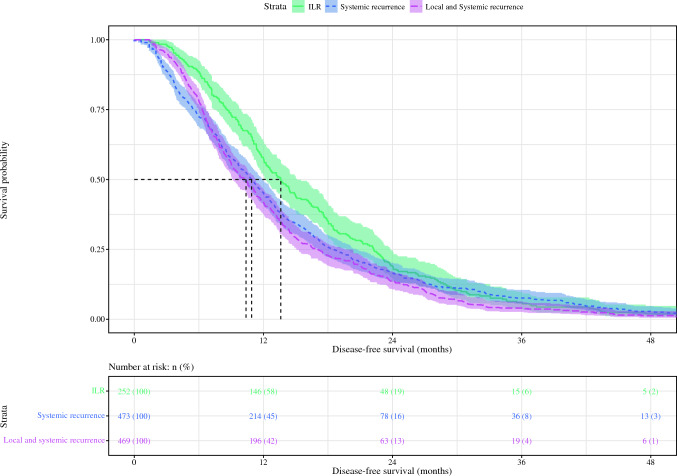
Kaplan–Meier comparing disease-free survival of patients with ILR with patients with (local and) systemic disease recurrence after resection of pancreatic ductal adenocarcinoma

**Fig. 2:**
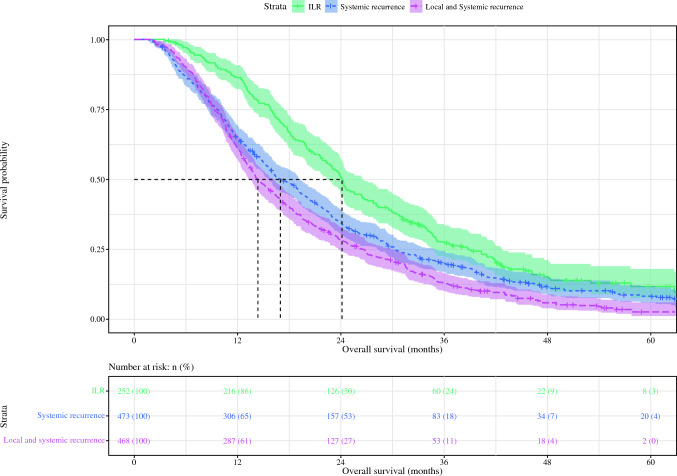
Kaplan–Meier comparing overall survival of patients with ILRwith patients with (local and) systemic disease recurrence after resection of pancreatic ductal adenocarcinoma

Patients with ILR had a DFS of 14 months (95% C 12–15 months) compared with 11 months (95% CI 10–12 months) in patients who developed systemic recurrence (*P* = 0.04) and 10 months (95% CI 9–11 months) in patients with synchronous local and systemic disease recurrence (*P* < 0.001; Figure 1). Patients who developed ILR had a higher OS of 24 months (95% CI 23–27 months) compared to 17 months (95% CI 16–19 months) in patients who developed systemic disease recurrence (*P* < 0.001) and 14 months (95% CI 13–16 months) in patients with synchronous local and systemic disease recurrence (*P* < 0.001; Fig. 2).

### Factors Associated with ILR

Since the proportionality assumption did not hold for adjuvant chemotherapy, this variable was included as a time-varying covariate. The best performing model included six covariates: resectability status (borderline resectable versus resectable, HR 1.42; 95% CI 1.03–1.96; *P* = 0.03; and locally advanced versus resectable, HR 1.11; 95% CI 0.68–1.82; *P* = 0.66), tumor location (head versus body/tail, HR 1.50; 95% CI 1.00–2.25; *P* = 0.05), vascular resection (yes versus no, HR 1.86; 95% CI 1.41–2.45; *P* < 0.001), perineural invasion (yes versus no, HR 1.47; 95% CI 1.01–2.13; *P* = 0.02), number of positive lymph nodes (continuous, HR 1.04; 95% CI 1.01–1.08; *P* = 0.02), and resection margin status (R1 < 1 mm versus R0 ≥ 1 mm, HR 1.64; 95% CI 1.25–2.14; *P* < 0.001; Table 3).

**Table 3:** Multivariable cause-specific Cox proportional hazard analysis to identify independent predictors of isolated local disease recurrence after resection of pancreatic ductal adenocarcinoma

	HR	95% CI	*P*
Resectability status			
Resectable	Ref	Ref	Ref
Borderline resectable	1.42	1.03–1.96	0.03
Locally advanced	1.11	0.68–1.82	0.66
Tumor location (head versus body/tail)	1.50	1.00–2.25	0.05
Vascular resection (yes versus no)	1.86	1.41–2.45	< 0.001
Perineural invasion (yes versus no)	1.47	1.02–2.13	0.04
Number of positive regional lymph nodes (continuous)	1.04	1.01–1.08	0.02
Resection margin status^a^			
R0 ≥ 1 mm	Ref	Ref	Ref
R1 < 1 mm	1.64	1.25–2.14	< 0.001

^a^Resection margin status was considered microscopically positive (R1 < 1 mm) if tumor cells were present within 1 mm of the closest resection margin, apart from the anterior surface

*95% CI* 95% confidence interval, *HR* Hazard ratio

### Prognostic Model

**Fig. 3:**
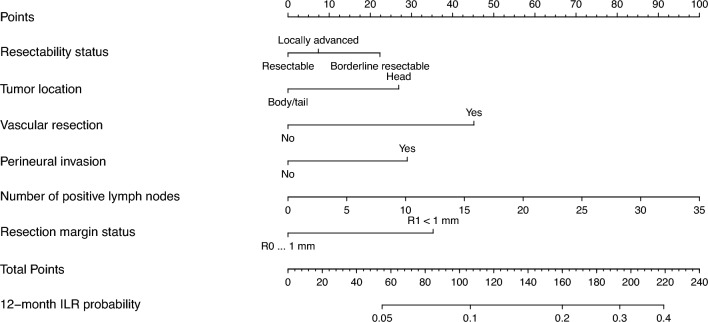
Nomogram to predict the probability of ILR 12 months after resection of pancreatic ductal adenocarcinoma

Risk scores were assigned to each predictor, with a total maximum score of 258 (Supplementary Table 2). The best predictive model had a C-index of 0.66 and calibration slope of 0.99 (Fig. 3, Supplementary Fig. 2).

## Discussion

This large, nationwide cohort study identified several predictive factors for ILR among patients after resection of PDAC. The online model based on the best performing combination of these factors included resectability status, location of the tumor, vascular resection, perineural invasion, number of positive regional lymph nodes, and resection margin status.

Literature on predictive factors for ILR is relatively scarce. Although R1 < 1 mm seems to be a prognostic factor based on the study by Groot et al. and our study, Jones et al. did not find a significant association with ILR.^5,10^ Interestingly, the anterior margin was not included in the determination of resection margin in the study by Groot et al. and our study, while Jones et al. did include this margin in the assessment.^5,10^ This resulted in a higher number of R1 resections in the latter study and suggests that a positive resection margin is only predictive of ILR when the anterior surface is disregarded.^10^ Moreover, the inclusion of factors, such as resection margin status, resectability status, the need for vascular resection, presence of perineural invasion, and a higher number of positive locoregional lymph nodes in the final model, indicates that ILR mainly occurs from tumors that are locally more advanced. In addition, ILR seems to occur from biologically more favorable tumors compared with tumors that result in synchronous local and systemic recurrence. This suggests that tumors that develop into local recurrences have a different tumor biology than tumors that tend to spread systemically. Future studies should aim at revealing the underlying biological differences that contribute to distinct patterns of recurrence.

Adjuvant chemotherapy was found to be associated with reduced occurrence of ILR in the meta-analysis by Tanaka et al. (*n* = 894), whilst this association was not seen in the study by Groot et al. (*n* = 692) and our study.^5,11^ However, the five studies included in the meta-analysis by Tanaka et al. to determine the prognostic value of adjuvant chemotherapy exhibit considerable heterogeneity. Both retrospective cohort studies and prospective randomized controlled trials (RCTs) were analyzed.^23–27^ In the two retrospective studies included, the effect of adjuvant chemotherapy on ILR was calculated on the basis of analyses, which were not designed to determine this outcome.^23,24^ In one of the three RCTs, adjuvant chemotherapy was compared with adjuvant chemoradiotherapy, which impeded evaluation of the true effect of adjuvant chemotherapy.^25^ Additionally, the remaining two RCTs did not find a significant correlation between adjuvant chemotherapy and ILR, which aligns with the results of Groot et al. and our study.^5,26,27^ Therefore, the prognostic value of adjuvant chemotherapy in light of ILR seems questionable at least. Regarding the type of adjuvant chemotherapy, Jones et al. found that patients who received adjuvant gemcitabine/capecitabine showed a reduced likelihood of developing ILR compared with receiving gemcitabine alone.^10^ In addition, the 5-year results of the PRODIGE 24/Canadian Cancer Trials Group PA6 demonstrated a similar proportion of patients with ILR after modified 5-fluorouracil, leucovorin, irinotecan, and oxaliplatin combination (mFOLFIRINOX) as after Gemcitabine monotherapy.^28^ Therefore, whether or not adjuvant chemotherapy is related to the development of ILR and whether it depends on the specific regimen received remains unclear and requires further investigation. However, Groot et al. did demonstrate that adjuvant chemoradiotherapy reduces the chance of developing ILR.^5^ This finding supports the hypothesis that ILR originates from residual microscopic tumor deposits, warranting further improvement of local therapies. Lastly, pathologic response to chemotherapy was not collected in this study but would be an interesting factor to investigate in future studies.

Patients with ILR after resection of PDAC demonstrate superior OS compared with patients with disease recurrence in the liver or at multiple sites and might specifically benefit from local ablative treatment.^7^ Over the past years, image-guided stereotactic body radiation therapy (SBRT) gained interest as potential local treatment for ILR, since it allows higher dose administration to the intended target area while sparing the surrounding organs.^29–33^ Additionally, magnetic resonance guided RT (MRgRT) with daily online adaptive treatment planning and tumor gating using continuous cine MR-images significantly enhances the visibility of both tumor and organs at risk.^34–37^ These advancements have made it possible to deliver higher biologically equivalent radiation doses to pancreatic lesions, potentially increasing treatment effiveness.^38,39^ Currently, the value of additional SBRT in patients with ILR is investigated in the nationwide randomized controlled ARCADE trial (NCT04881487).^40^ Besides the combination of SBRT and chemotherapy, SBRT and immunotherapy might be promising as well for the treatment of ILR. Radiotherapy might trigger tumor-associated antigens and upregulation of immune checkpoints, which are targeted by immunotherapy.^41,42^ Zhu et al. combined CT-guided SBRT with pembrolizumab and trametinib and compared it with CT-guided SBRT and gemcitabine.^43^ They have observed improved survival in patients with local recurrence after resection of PDAC that received pembrolizumab and trametinib, although this coincided with increased serious adverse events. The optimal combination of local and systemic treatment for ILR, however, is yet to be determined.

Before disease dissemination might occur, depriving patients’ opportunity to receive local ablative treatment, early identification of ILR through recurrence-focused follow-up with imaging at certain intervals seems important. Within the Netherlands, it is uncommon to conduct recurrence-focused follow-up imaging after PDAC resection, following recommendations in Dutch and European guidelines.^19,44,45^ Nevertheless, if routine postoperative imaging enables early detection of disease recurrence, this could potentially enhance timely treatment, which is anticipated to have a positive impact on survival. This hypothesis is currently being investigated in the RADAR-PANC trial (NCT04875325).

The detection of ILR on follow-up imaging often poses a challenging task. Distinguishing recurrent tumor tissue from postoperative fibrosis on postoperative imaging can be difficult. As a result, repetitive imaging is frequently required to assess whether the lesion grows over time, indicating tumor recurrence.^8,9^ Promising developments in computer science might provide a solution for this challenge, as artificial intelligence (AI) techniques have shown to be particularly helpful for imaging evaluation. For example, AI finds great utility in highlighting suspicious regions in imaging and classifying abnormalities as benign or malignant.^46^ The IMPACT consortium (NCT06055010) focuses on the development of an AI algorithm that can help clinicians to differentiate between postoperative fibrosis and pancreatic cancer recurrence, aiming to enhance an early and more accurate diagnosis of ILR.^47^ Until diagnostic accuracy is approved, however, the prognostic model developed in this study can be used to identify patients with a higher risk of developing ILR, which might be informative when evaluating patients’ follow-up imaging.

Main strengths of this study include the largest study population so far to investigate ILR specific risk factors, with subsequent integration of selected factors into a comprehensive online available prognostic model. However, the results of this study should be interpreted with acknowledgement of several limitations. First, baseline and perioperative data were collected in a prospective manner, while data on follow-up and recurrence were obtained retrospectively from the patients’ records. Second, the proportion of patients with ILR of 21% found in this study might be an underestimation as imaging is generally only performed in patients with symptoms of disease recurrence according to the Dutch national guidelines.^19^ This might have caused patients who suffered from initial ILR but developed systemic metastases before the diagnosis of disease recurrence to be misclassified into the group with local and systemic disease recurrence. Third, histological evidence of disease recurrence was only obtained in a minority of patients. When histological evidence was absent, presence of recurrence and corresponding recurrence location was based on consensus of a multidisciplinary meeting based on imaging and CA 19-9 levels. In case of ILR, this might mean that a small number of patients with postoperative fibrosis have wrongfully been classified as ILR. Lastly, borderline resectable disease is awarded more points than locally advanced disease in the nomogram, which seems unusual. However, the subgroup of patients with locally advanced disease was relatively small (only 7%), which could impede robustness of findings. Additionally, only patients with locally advanced disease who underwent pancreatic cancer resection were included. These patients reflect the best patients amongst the total group with locally advanced disease, as they responded well to neoadjuvant systemic therapy, which downsized their tumor, making them eligible for tumor resection. In contrast, most patients with borderline resectable disease in this study underwent upfront resection, as neoadjuvant therapy was only administered as part of a clinical trial during the study period.^17,18^ This is also reflected by their higher R1 resection margin rate (66% versus 44% in patients with locally advanced disease). As microscopically irradical resections (R1 < 1 mm) were associated with a higher probability of developing ILR, the higher number of points awarded to borderline disease might be explained by a higher probability of developing ILR based on the higher rate of microscopically irradical resections related to the difference in neoadjuvant treatment and patient selection.^5^

To conclude, multiple factors associated with ILR of PDAC have been identified in this nationwide, observational cohort study. The developed prognostic model, available at www.pancreascalculator.com, can be helpful to identify patients with a higher risk of developing ILR. This can be informative to healthcare professionals when evaluating patients’ postoperative imaging and for patient counseling.

## Supplementary Information

Below is the link to the electronic supplementary material.

Supplementary file1 (DOCX 102 kb)
