# Supplementary material for: Prediction of Isolated Local Recurrence After Resection of Pancreatic Ductal Adenocarcinoma: A Nationwide Study
Source: Ann Surg Oncol. 2024 Jun 27;31(12):8264–75. doi: 10.1245/s10434-024-15664-4 (PMC11467030; doi:10.1245/s10434-024-15664-4)
Supplement: Supplementary file 1 — Supplementary file1 (DOCX 102 kb) [file 10434_2024_15664_MOESM1_ESM.docx]

**Supplemental Figures and Tables**

***Supplemental Figure 1.*** *Flow diagram of inclusion process*

Resection of PDAC 2014-2019

Assessed for eligibility (n = 1909)

Excluded (n = 216)

- Complication-related mortality within 90 days postoperative (n = 83)
- Macroscopically irradical resection or unknown resection margin status (n = 65)
- Unknown recurrence status (n = 18)
- Recurrence with unknown location (n = 50)

Included (n = 1693)

***Supplemental Figure 2.*** *Calibration plot of final predictive model*

*
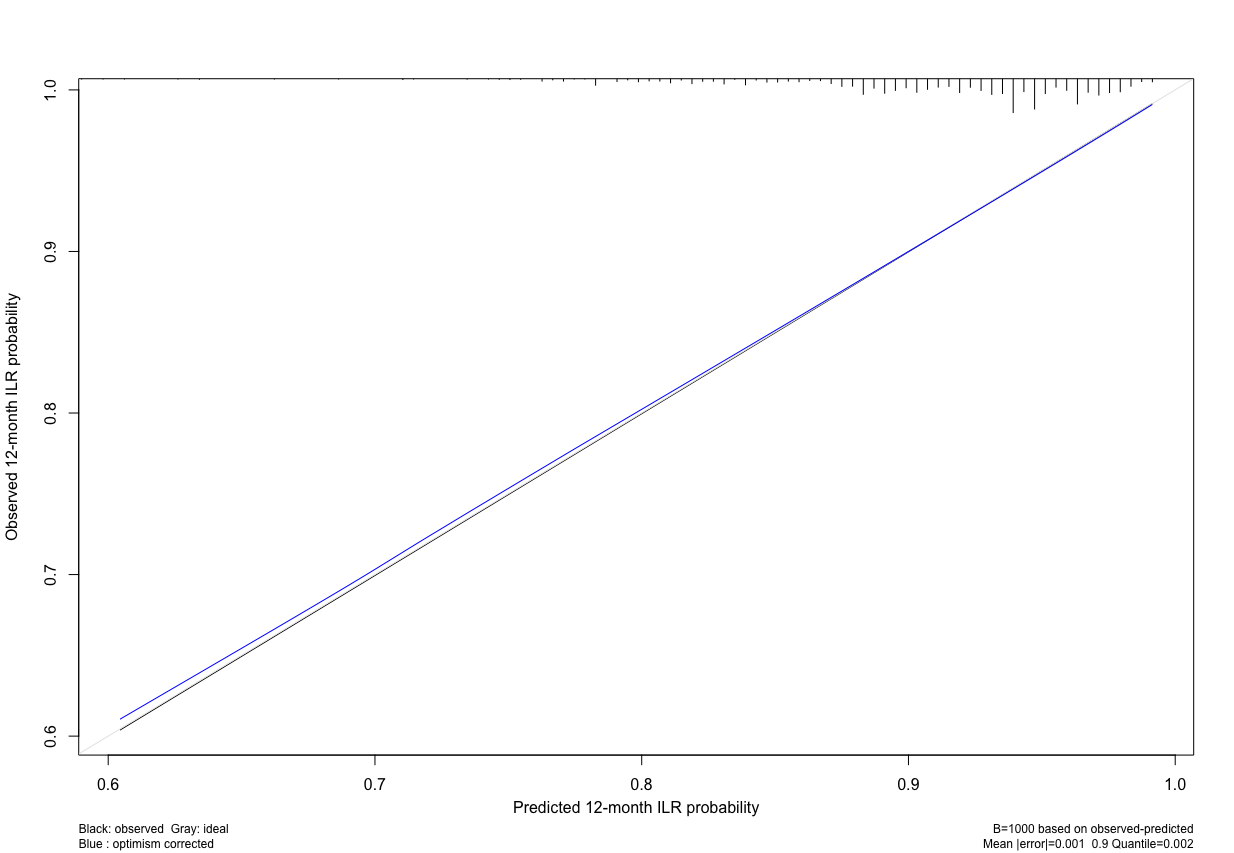
*

| ***Supplemental Table 1.*** *Descriptive statistics comparing patients with isolated local pancreatic ductal adenocarcinoma recurrence to patients with local and systemic recurrence* | | | |
| --- | --- | --- | --- |
|  | **ILR**  **(n = 252)** | **Local and systemic**  **(n = 469)** | **P** |
| Age at diagnosis, mean (SD), years | 66 (10) | 67 (10) | 0.49 |
| Male sex, n (%) | 136 (54) | 244 (52) | 0.62 |
| BMI, mean (SD), kg/m^2^ | 25 (4) | 25 (4) | 0.92 |
| CACI, mean (SD) | 3 (2) | 3 (2) | 0.75 |
| Resectability, n (%) |  |  | 0.22 |
| Resectable | 175 (70) | 355 (76) |  |
| Borderline resectable | 56 (22) | 81 (17) |  |
| Locally advanced | 21 (8) | 33 (7) |  |
| Neoadjuvant therapy, n (%) | 42 (17) | 66 (14) | 0.35 |
| Completed neoadjuvant therapy^a^, n (%) | 37 (71) | 61 (92) | 0.53 |
| Preoperative serum CA 19-9 level, median (IQR), U/mL | 157 (34-514) | 179 (48-593) | 0.06 |
| Tumor location, n (%) |  |  | 0.09 |
| Head | 226 (90) | 397 (85) |  |
| Body/tail | 26 (11) | 72 (15) |  |
| Vascular resection, n (%) | 99 (39) | 145 (31) | 0.03 |
| Tumor stage 8^th^ AJCC edition, n (%) |  |  | < 0.01 |
| T1 | 22 (9) | 52 (11) |  |
| T2 | 176 (70) | 264 (56) |  |
| T3 | 49 (20) | 147 (31) |  |
| T4 | 5 (2) | 6 (1) |  |
| Tumor differentiation, n (%) |  |  | 0.02 |
| Well/moderate | 192 (76) | 323 (69) |  |
| Poor | 60 (24) | 146 (31) |  |
| Perineural invasion, n (%) | 217 (86) | 405 (86) | 0.89 |
| Lymphovascular invasion, n (%) | 139 (55) | 334 (71) | < 0.001 |
| Lymph node status 8^th^ AJCC edition, n (%) |  |  | 0.12 |
| N0 | 69 (27) | 116 (25) |  |
| N1 | 103 (41) | 168 (36) |  |
| N2 | 80 (32) | 185 (39) |  |
| Resection margin status^b^, n (%) |  |  | 0.31 |
| R0≥1 mm | 103 (41) | 210 (45) |  |
| R1<1 mm | 149 (59) | 259 (55) |  |
| Adjuvant chemotherapy, n (%) | 170 (67) | 264 (56) | < 0.01 |
| Completed adjuvant chemotherapy^a^, n (%) | 120 (71) | 166 (63) | < 0.01 |
| Use of imaging procedures during follow-up^c^, n (%) |  |  | 0.02 |
| None/non-standardized | 195 (78) | 390 (83) |  |
| Standardized | 54 (22) | 68 (14) |  |
| Percentages may not add up to 100 because of rounding and missing data.  ^a^ (Neo)adjuvant therapy was considered completed in case 80% of the planned number of cycles was received by the patient.  ^b^ Resection margin status was considered microscopically positive (R1<1 mm) if tumor cells were present within 1 mm of the closest resection margin, apart from the anterior surface.  ^c^ Postoperative imaging could have been performed in a standardized fashion at set intervals, or when indicated by clinical symptoms.  AJCC: American Joint Committee on Cancer; BMI: body mass index; CA 19-9: Carbohydrate Antigen 19-9; CACI: Charlson Age-adjusted Comorbidity Index; IQR: Interquartile range; P: probability-value; SD: standard deviation. | | | |

| ***Supplemental Table 2****. Risk table of the final predictive model for isolated local recurrence after resection of pancreatic ductal adenocarcinoma* | |
| --- | --- |
|  | Risk score |
| Resectability status |  |
| Resectable | 0 |
| Borderline resectable | 22 |
| Locally advanced | 7 |
| Tumor location |  |
| Body/tail | 0 |
| Head | 27 |
| Vascular resection |  |
| No | 0 |
| Yes | 45 |
| Perineural invasion |  |
| No | 0 |
| Yes | 29 |
| Number of positive regional lymph nodes |  |
| 0 | 0 |
| 5 | 14 |
| 10 | 29 |
| 15 | 43 |
| 20 | 57 |
| 25 | 71 |
| 30 | 86 |
| 35 | 100 |
| Resection margin status^a^ |  |
| R0≥1 mm | 0 |
| R1<1 mm | 35 |
| ^a^ Resection margin status was considered microscopically positive (R1<1 mm) if tumor cells were present within 1 mm of the closest resection margin, apart from the anterior surface. | |
